# Patterns of Chlamydia trachomatis and Neisseria gonorrhoeae in different anatomical sites among Pre-Exposure Prophylaxis (PrEP) users in Brazil

**DOI:** 10.1186/s12879-024-09144-z

**Published:** 2024-02-26

**Authors:** Marcela Antonini, Mario Vianna Vettore, Anita Øgård-Repål, Daniel de Macêdo Rocha, Karyanna Alves de Alencar Rocha, Henrique Ciabotti Elias, Felipe Barufaldi, Rodrigo Carvalho Santana, Elucir Gir, Bruno Spire, Renata Karina Reis

**Affiliations:** 1 University of São Paulo (USP), Ribeirão Preto College of Nursing, Ribeirão Preto, São Paulo, Brazil; 2 Department of Health and Nursing Sciences, University of Agder (UiA), Kristiansand, Vest- Agder, Norway; 3 University of São Paulo, Ribeirão Preto Medical School, Ribeirão Preto, São Paulo, Brazil; 4 Aix-Marseille Université, Marseille, France; 5 Department of Dentistry and Oral Health, Aarhus University, Aarhus, Denmark

**Keywords:** Pre-exposure Prophylaxis, HIV/aids, HIV prevention, Sexually transmitted infections, Chlamydia trachomatis, Neisseria gonorrhoeae

## Abstract

**Background:**

The presence of untreated sexually transmitted infections (STIs) significantly increases the chance of acquiring HIV. In Brazil, testing for Chlamydia trachomatis (CT) and Neisseria gonorrhoeae (NG) among Pre-Exposure Prophylaxis (PrEP) users is insufficient, and syndromic treatment is a priority in clinical practice. Multi-site testing for CT/NG improves thescreening of asymptomatic cases and ensures timely treatment. Therefore, it is essential for HIV prevention. This study aims to test the importance of two-site testing for better screening of these pathogens and to determine whether the presence of symptoms is an indicator of CT/NG infection.

**Methods:**

This is a cross-sectional study carried out in four public infectious diseases clinics in São Paulo State, Brazil between January of 2022 and March of 2023. All participants had an anal swab and a first-pass or mid-stream urine collected for CT/NG analysis by Polymerase chain reaction (PCR). Data about sociodemographic, sexual behavioural and clinical aspects were collected. Pathway analysis was used to examine the direct and indirect relationships between variables according to the theoretical model.

**Results:**

We screened 171 PrEP users which had two samples collected, resulting in 342 samples. Comparing the anatomic sites, the urine samples showed lower sensitivity for CT and NG than anal samples. Gonorrhoea was directly linked to lower age (β= -0.161, *p* = 0.001). Time of PrEP use was directly associated with CT infection (β = 0.202; *p* = 0.042) and inversely associated with dysuria (β= -0.121, *p* = 0.009). Lower occurrence of yellow-green secretion was linked to detection of CT (β= -0.089, *p* = 0.005) and NG (β= -0.048, *p* = 0.002) infections. Foul-smelling discharge was directly associated with CT (β = 0.275, *p* = 0.004) and NG (β = 0.295, *p* = 0.037) infection.

**Conclusion:**

The symptoms are a bad indicator of CT and NG infection, and the screening must be done in more than one site since most of the positive results would be missed if only urines were tested. In the case of testing only one anatomical site, specifically the urethra, the CT/NG incidence and prevalence would be underestimated. The two-sites testing improves detection rates of CT/NG, and PrEP follow-up benefits people offering STI testing.

## Background

Pre-exposure prophylaxis (PrEP) is a safe and effective approach to HIV prevention when high adherence is maintained among individuals at substantial risk of infection. It was incorporated in into the Brazilian Unified Health System in 2017 and has become a notable resource in the spectrum of combined prevention and results in greater demand and integration with health services for testing and counselling. According to the World Health Organization (WHO), HIV prevention must also consider the high risk of other Sexually Transmitted Infections (STIs), as their presence and diagnostic or therapeutic delay can trigger molecular changes, that induces an inflammatory response, which might interfere withthe effectiveness of PrEP, increasing the risk of HIV infection by up to 10 times [1, 2].

From this perspective, *Chlamydia trachomatis* (CT) and *Neisseria gonorrhoeae* (NG) infections stand out. These infections are often asymptomatic and may involve a broad clinical spectrum, presenting multiple sites of infection. If untreated, they can result in pelvic inflammatory disease, maternal-fetal complications, and infertility [3]. The literature has shown a considerable prevalence among individuals who started PrEP and a significant incidence among persistent users. The epidemiological impact of these infections must be understood in light of the potential for ascertainment bias, as testing asymptomatic individuals is often neglected by some optimal PrEP monitoring practices [3, 4].

Tracking, diagnosing, and treating positive cases of chlamydia and gonorrhoea in multiple sites should be considered key elements for successful HIV prevention. In Brazil, the current national protocol recommends the Polymerase Chain Reaction (PCR) as the first option for CT/NG screening, and it should be performed every six months among PrEP users [5]. These strategies reflect and provide the opportunity to offer comprehensive tests in specialized services to identify, in multiple sites, the presence of asymptomatic cases, as well as to promote adequate treatment and break the transmission chain [6]. Despite these recommendations, the implementation of clinical protocols for CT and NG screening, and the structuring of services capable of facing the synergistic epidemics of HIV and other STIs are challenging conditions in Brazil [7]. Moreover, high costs, low professional training, and structural precariousness to meet the demands of laboratory work are barriers to offering these tests.

In Brazil, few studies have addressed the incorporation of CT/NG screening into PrEP services in a real environment and their detection in different anatomical sites. ​​Based on this, this study contributes with evidence on the prevalence of CT/NG in Brazil. This study aimed to test the importance of two-site testing for better screening of these pathogens and to test the presence of symptoms as an indicator for CT/NG infection.

## Method

This is a cross-sectional study carried out in four public infectious diseases clinics in the city of Ribeirão Preto, São Paulo, Brazil between January of 2022 and March of 2023. All participants were offered tests for CT and NG screening at two anatomical sites (urethra and anorectum). All of them had a first-pass urine and anal swab collected for molecular tests by the Abbott commercial kits. The Abbott RealTime CT/NG assay is a polymerase chain reaction (PCR) assay for the direct, qualitative detection of the plasmid DNA for *C. trachomatis* and the genomic DNA of *N. gonorrhoeae*. The PCR was carried out into 45-well plates, of which two wells were used for internal controls. The internal controls were provided in the combo and showed a 91.3% positive agreement for CT and 97.8% for NG quality [8].

### Recruitment, inclusion criteria and data collection

A convenience sample of PrEP users aged at least 16 years, and with active sex life was recruited. Participants were invited to participate in the study in private rooms after their medical appointments for PrEP follow-up, due to sensitive questions. After obtaining written consent, participants responded to a questionnaire about sociodemographic characteristics, sexual behaviours, previous STIs and self-reported symptoms of chlamydia and/or gonorrhoea infections. After the interview, they had the samples collected for CT and NG screening.

Data was collected by registered nurses who were members of the research group. They were previously trained to conduct the interviews and to handle the samples from the clinics to the laboratory. Participants with positive results for CT and/or NG were treated through the Brazilian Unified Health System according to the national protocol for the treatment of STIs [9]. We excluded returning participants in this analysis to avoid the bias of multiple visits and testing of the same individuals.

### Sample size calculation

A sample of 171 people was estimated to detect a minimum effect size of 0.1 with a power of 80% and 0.05 level of significance (α = 0.05), considering a response rate of 70% to test the direct and indirect effects of a complex model with 12 observed variables through a pathway analysis.

### Measures

Sociodemographic data - sex, age, gender, skin colour, schooling (fundamental, high school and undergraduate), maritage status (single, married, divorced) and sex worker (yes/no).

Behavioural data– All the sexual activities were measured considering the last 30 days. It included: sexual preference (have sex with men, women, both), number of sexual partners (continuous), type of partners (casual, stable, both), sex in group (yes/no), anal sex (yes/no), vaginal sex (yes/no), oral sex (yes/no) and anilingus (yes/no). All the variables about sex activity considered the insertive and/or receptive anal practice. So, anal sex, for example, wasn’t measured only by receptive position. Further, data about alcohol consumption (never, regularly, only before having sex) and drug use (never, regularly, chemsex) was collected considering the last six months.

Clinical data– anti-HIV serology of the partner (positive, negative, do not know), previous STI in the past 6 months (syphilis, chlamydia, gonorrhea, human papillomavirus - HPV, viral hepatitis, Genital Herpes, Monkeypox), completed treatment of previous STI (yes/no), current symptoms including in the day that participated of the study (urethral, vaginal or anal discharge with yellow/greenish secretion, foul-smelling discharge, dysuria), PCR results for CT/NG infections for each anatomical site (urethral and anorectum).

### Outcome

The positive PCR for CT or NG was considered the outcome and the golden standard in this study. We evaluated how many diagnoses would have been missed if only one site was tested.

### Data analysis

The distribution of the variables for the total sample and according to positive detection for Chlamydia and Gonorrhoea were reported using means and standard deviations (continuous variables) and proportions (categorical variables).

The prevalence of site-specific and multisite infections of chlamydia and gonorrhoea were calculated. We evaluated how many diagnoses would have been missed if only one site had tested and analyzed the symptoms as an indicator of CT or NG infection. Participants who had tested positive for infection at two anatomical sites were defined as multisite infection. The proportion of single-site and multisite chlamydia and gonorrhoea infections at each anatomical site was described.

Sensitivities for the presence of any symptom approach were calculated against the result of any positive PCR samples as the gold standard. The χ2 test was used to compare the validity indices, including positive predict value, negative predict value, sensitivity and specificity between CT and NG according to the presence of symptoms and different anatomical sites.

**Fig. 1:**
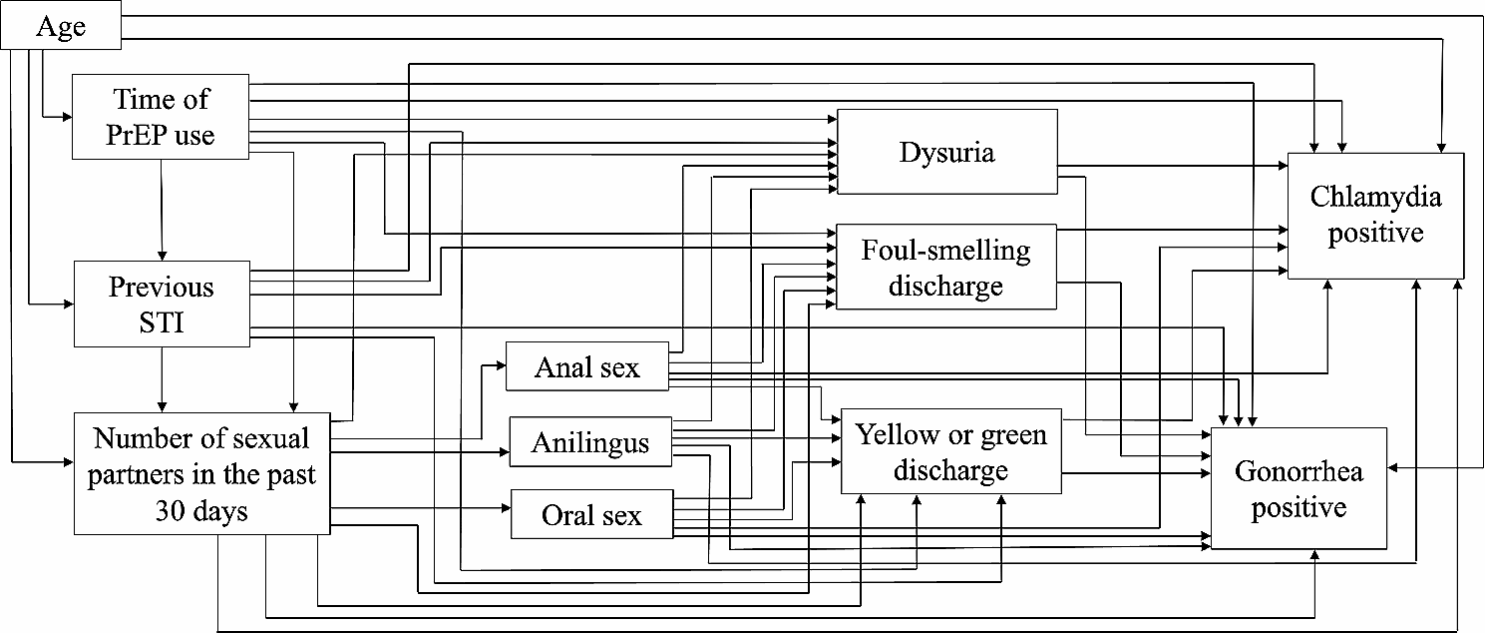
Full theoretical model, showing hypothesised paths between variables

Pathway analysis was used to examine the direct and indirect relationships between variables according to the theoretical model. Initially, the full model (Fig. 1) was tested for identification, evaluation and adjustment of the fit indices. Then, non-significant paths considering *p* value > 0.05 were removed to obtain a parsimonious model. The paths between the three variables representing clinical symptoms (malodorous, discharge and dysuria) and positive detection for CT and NG were retained in the model according to the central hypotheses of the study.

The parsimonious model was interpreted using the standardised direct and indirect effects. The former represented a direct path from one variable to another while the latter was used to evaluate mediation, that is when the association between two variables was mediated by another variable. The models were estimated using the maximum likelihood method and bootstrap procedure to estimate the 95% confidence intervals through resampling nine hundred samples from the original dataset (CIs) [10].

The adequacy of the full and parsimonious models was assessed using the following fit indexes and threshold values: χ2 < 3.0, standardised rootmean-square residual (SRMR) ≤ 0.08, root-mean-square error of approximation (RMSEA) ≤ 0.06, comparative fit index (CFI) ≥ 0.90 goodness of fit index (GFI) ≥ 0.90, and Tucker-Lewis index (TLI) ≥ 0.90 [11].

Descriptive analyses were carried out using SPSS software (Statistical Package for Social Sciences) version 29.0. Confirmatory factorial analysis and Structural Equation Modelling (SEM) were conducted using AMOS SPSS version 29.0. Mainly we tested the importance of multisite testing for better screening of these pathogens and the presence of symptoms as an indicator for CT/NG infection.

### Ethical aspects

All the data was collected after the participants’ written consent. This study was carried out according to the Helsinki Declaration. The project was approved by the Humans Ethical prepotent institution under number 5.111.296.

## Results

The final sample was composed of 171 (100%) participants which had two biological samples collected (urine and anal swab), resulting in 342 samples. There were 15.7% (27/171) participants who tested positive for at least one pathogen at any site; 11.7% (20/171) were positive for CT and 7.0% (12/171) for NG.

Regarding the profile of participants, the median age of participants was 34.15 (IQR 20–73) years. Eight-four% (144/171) participants were single or divorced; 93.4% (160/171) were men, 61.4% (105/171) white, 68.4% (117/171) graduated, and 85.4% (146/171) referred to had sex only with men. Table 1 shows the distribution of CT and NG cases according to the sociodemographic data, independently of the anatomical site. It is possible to see that the cases of CT and NG were more frequent among those with higher education.

**Table 1:** Profile of PrEP users and the distribution of CT/NG infection cases. Brazil, 2023

Variables		Total	CT infection**		NG infection**	
			negative	positive	negative	positive
**Sex**	Female	11 (6.4)	11 (6.4)	-	11 (6.4)	-
	Male	160 (93.6)	140 (81.9)	20 (11.7)	148 (86.5)	12 (7.0)
**Skin colour**	White	105 (61.4)	92 (53.8)	13 (7.6)	100 (58.5)	05 (2.9)
	Black	26 (15.2)	24 (14.0)	02 (1.2)	20 (11.7)	06 (3.5)
	Others	40 (23.4)	35 (20.5)	05 (2.9)	39 (22.8)	01 (0.6)
**Gender**	Cisgender women	11 (6.4)	11 (6.4)	-	11 (6.4)	-
	Cisgender men	152 (88.9)	134 (78.4)	18 (10.5)	140 (81.9)	12 (7.0)
	Transgender women	08 (4.7)	06 (3.5)	02 (1.2)	8.0 (5.0)	-
**Marital status**	Single or divorced	144 (84.2)	126 (73.7)	18 (10.5)	132 (77.2)	12 (7.0)
	Married / stable union	27 (15.8)	25 (14.6)	02 (1.2)	27 (15.8)	-
**Schooling**	Primary school	06 (3.5)	05 (2.9)	01 (0.6)	06 (3.8)	-
	High school	48 (28.1)	44 (25.7)	04 (2.3)	44 (25.7)	04 (2.3)
	Graduated	117 (68.4)	102 (59.6)	15 (8.8)	109 (63.7)	08 (4.7)
**Sex worker**	No	166 (97.1)	147 (86.0)	19 (11.1)	154 (90.1)	12 (7.0)
	Yes	05 (2.9)	04 (2.3)	01 (0.6)	05 (2.9)	-

** independently of the anatomical site

Regarding the behavioural data, Table 2 shows that 53.8% of participants had casual partners (92/171) and 14.6% had casual and stable partners concomitantly (25/171). The mean number of partners was 7.28 (IQR 1-300, Std error: 1.916); 94.7% (162/171) participants had condomless oral sex; 66.6% referred to alcohol consumption (88/171 regularly, 26/171 only before having sex), and 12.3% (21/171) chemsex.

**Table 2:** Distribution of CT/NG infection according to the behavioural variables. Brazil, 2023

Variables		Total	CT infection**		NG infection**	
			negative	positive	negative	positive
**Usually have sex with**	Only women	02 (1.2)	02 (1.2)	-	02 (1.2)	-
	Only men	146 (85.4)	130 (76.0)	16 (9.4)	137 (80.1)	09 (5.3)
	Both	23 (13.5)	19 (11.1)	04 (2.3)	20 (11.7)	03 (1.8)
**Type of partner**	Stable	54 (31.6)	50 (29.2)	04 (2.3)	53 (31.0)	01 (0.6)
	Casual	92 (53.8)	79 (46.2)	13 (7.6)	82 (48.0)	10 (5.8)
	Both	25 (14.6)	22 (12.9)	03 (1.8)	24 (14.0)	01 (0.6)
Oral sex*	No	09 (5.3)	09 (5.3)	-	09 (5.3)	-
	Yes	162 (94.7)	142 (63.0)	20 (11.7)	150 (87.7)	12 (7.0)
Vaginal sex*	No	150 (87.7)	133 (77.8)	17 (9.9)	138 (80.7)	12 (7.0)
	Yes	21 (12.3)	18 (10.5)	03 (1.8)	21 (12.3)	-
Anal sex*	No	16 (9.4)	16 (9.4)	-	16 (9.4)	-
	Yes	155 (90.6)	135 (78.9)	20 (11.7)	143 (83.6)	12 (7.0)
Anilingus*	No	52 (30.4)	48 (28.1)	04 (2.3)	49 (28.7)	03 (1.8)
	Yes	119 (69.6)	103 (60.2)	16 (9.4)	110 (64.3)	09 (5.3)
Sex in group*	No	166 (97.1)	147 (86.0)	19 (11.1)	154 (90.1)	12 (7.0)
	Yes	05 (2.9)	04 (2.3)	01 (0.6)	05 (2.9)	-
**Alcohol consumption in the last 6 months**	Never	57 (33.3)	50 (29.2)	07 (4.1)	53 (31.0)	04 (2.3)
	Regularly	88 (51.5)	77 (45.0)	11 (6.4)	81 (47.4)	07 (4.1)
	Only for sex	26 (15.2)	24 (14.0)	02 (1.2)	25 (14.6)	01 (0.6)
**Drug consumption in the last 6 months**	Never	133 (77.8)	119 (69.6)	14 (8.2)	123 (71.9)	10 (5.8)
	Regularly	17 (9.9)	13 (7.6)	04 (2.3)	16 (9.4)	01 (0.6)
	Chemsex	21 (12.3)	19 (11.1)	02 (1.2)	20 (11.7)	01 (0.6)

*in the past 30 days; ** independently of the anatomical site

In Table 3, the infection by CT and NG independently of the anatomic site was kept as an outcome. However, it is possible to observe the distribution of positive cases according to clinical variables, as well as the detection of pathogens by each anatomical site. At this moment, the coinfection cases were not described.

Of all, 14.6% (25/171) of the participants were diagnosed with any STI in the past 6 months; 10.5% (18/171) reported having been diagnosed with CT or NG, and all of them completed the treatment in the past. 7.6% (13/171) participants had any STI symptoms when tested and, only 2.3% (04/171) of them had a positive PCR result of CT and 1.8% (03/171) of NG. A couple of them reported any classic symptoms suggestive of CT or NG infection. Three and half percent (06/171) had foul-smelling secretion; 2.3% (04/171) had yellow or green discharge, and 5.8% (10/171) had dysuria when tested. STI symptom frequency did not differ significantly between those who had CT (2.3%; 04/171) vs. NG (1.8%; 03/171).

Notably, the urine samples had a lower detection of pathogens than the anal samples. Only 2.9% (05/171) of urine samples had a positive result; of which 1.2% (02) were positive for CT only and 1.8% (03) for multipathogens. The anal samples showed a higher frequency of screening for any pathogen (14.61%; 25/171); in which 8.8% (15/171) were positive for CT, 5.3% (09/171) for NG, and 1.8% (03/171) for multipathogens. Almost 13% (12.9%; 22) of infections would have been missed and gone untreated if only urine samples had been collected.

**Table 3:** Distribution of CT/NG cases according to the clinical variables in PrEP users. Brazil, 2023

Variables		Total	CT infection**		NG infection**	
			negative	positive	negative	positive
**Partner living with HIV?**	No	67 (39.2)	64 (37.4)	03 (1.8)	63 (36.8)	04 (2.3)
	Yes	45 (26.3)	40 (23.4)	05 (2.9)	43 (25.1)	02 (1.2)
	Do not know	59 (34.5)	47 (27.5)	12 (7.0)	53 (31.0)	06 (3.5)
**Had any STI in the last 6 months**	No	146 (85.4)	131 (76.6)	15 (8.8)	137 (80.1)	09 (5.3)
	Yes	25 (14.6)	20 (11.7)	05 (2.9)	22 (12.9)	03 (1.8)
**Diagnosed with CT or NG in the last 6 months**	No	153 (89.5)	136 (79.5)	17 (9.9)	143 (83.6)	10 (5.8)
	Yes	18 (10.5)	15 (8.8)	03 (1.8)	16 (9.4)	02 (1.2)
Had any symptom when was tested*	No	158 (92.4)	140 (81.9)	18 (10.5)	148 (86.5)	10 (5.8)
	Yes	13 (7.6)	11 (6.4)	02 (1.2)	11 (6.4)	02 (1.2)
Yellow or green discharge*	No	167 (97.7)	148 (86.5)	19 (11.1)	156 (91.2)	11 (6.4)
	Yes	04 (2.3)	03 (1.8)	01 (0.6)	03 (1.8)	01 (0.6)
Foul-smelling discharge*	No	165 (96.5)	147 (86.0)	18 (10.5)	155 (90.6)	10 (5.8)
	Yes	06 (3.5)	04 (2.3)	02 (1.2)	04 (2.3)	02 (1.2)
Dysuria*	No	161 (94.2)	142 (83.0)	19 (11.1)	151 (88.3)	10 (5.8)
	Yes	10 (5.8)	09 (5.3)	01 (0.6)	08 (4.7)	02 (1.2)
**Had at least one pathogen detected in the study**	Negative	144 (84.2)	144 (84.2)	-	144 (84.2)	-
	Positive	27 (15.8)	07 (4.1)	20 (11.7)	15 (8.77)	12 (7.0)
**Urine sample**	Negative	166 (97.1)	151 (88.3)	15 (8.8)	157 (91.8)	09 (5.3)
	Positive	05 (2.9)	-	05 (2.9)	02 (1.2)	03 (1.8)
**Anal sample**	Negative	146 (85.4)	144 (84.2)	02 (1.8)	145 (84.8)	01 (0.6)
	Positive	25 (14.6)	07 (4.1)	18 (10.5)	14 (8.2)	11 (6.4)
**Prevalence of site-specific infections**	Negative	144 (84.2)	144 (84.2)	-	144 (84.2)	-
	Urine only	02 (1.2)	-	02 (1.2)	01 (0.6)	01 (0.6)
	Anal only	22 (12.9)	07 (4.1)	15 (8.8)	13 (7.6)	09 (5.3)
	Both	03 (1.8)	-	03 (1.8)	01 (0.6)	02 (1.2)

*in the past 30 days, including the day was tested; ** independently of the anatomical site

**Fig. 2:**
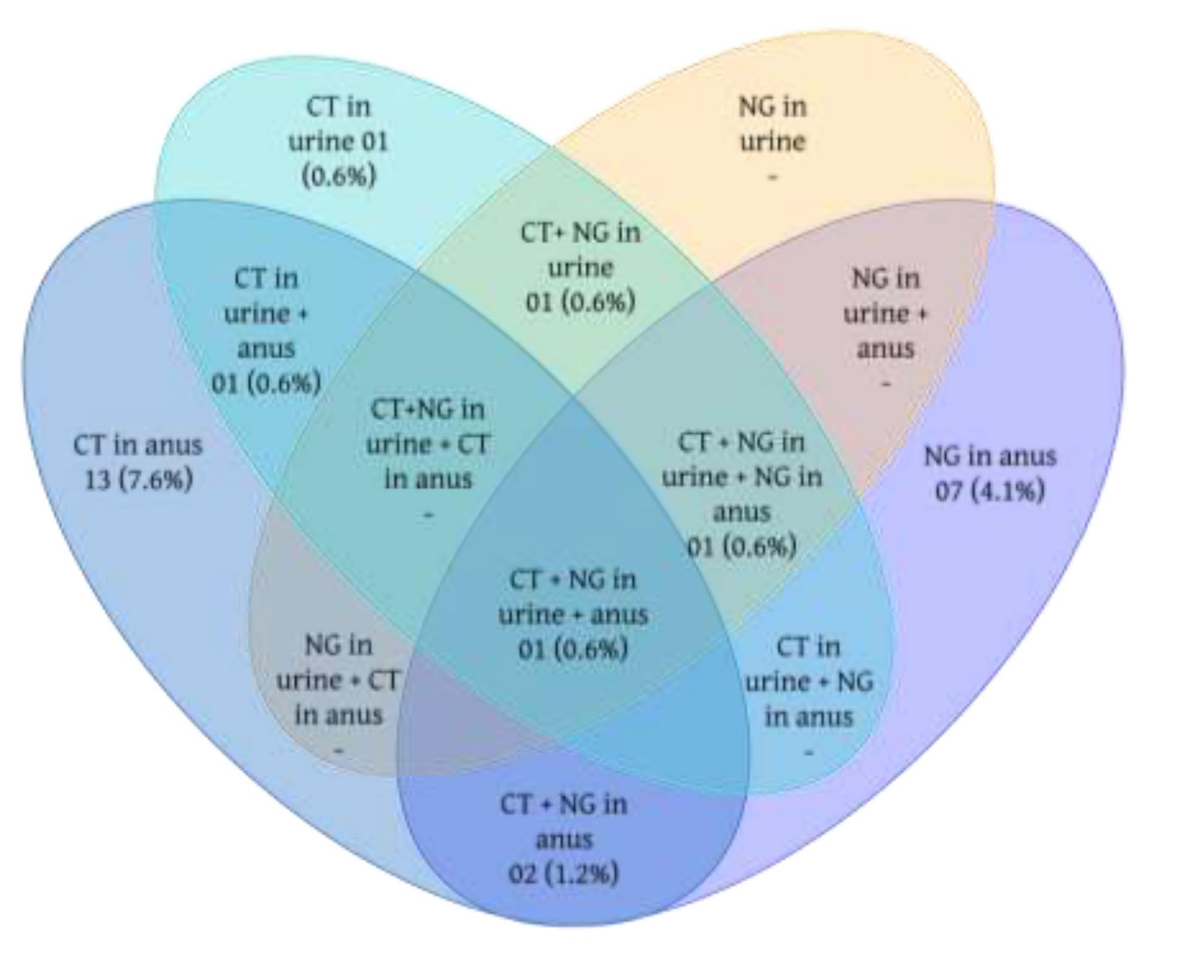
Distribution of *Chlamydia trachomatis* (CT) and *Neisseria gonorrhoea* (NG) according to the anatomical site. Brazil, 2023

Further, there were five (1.2%) cases of coinfections. The Fig. 2 shows the distribution of CT and NG cases per sample considering the co-infection even in different anatomical sites.

Table 4 shows the validity indices of symptoms and single-site infection of CT and NG. The presence of symptoms had a 15.4% positive predictive value, 10.0% sensitivity for CT infection and 16.7% for NG. Comparing the anatomic sites, the urine samples showed lower sensitivity for CT/NG than anal samples. The anal samples showed a sensibility of 81.8% for CT and 85.7% for NG.

**Table 4:** Validity indices of symptoms and single-site infection of gonorrhoea and chlamydia at each specific anatomical site, Brazil, 2023

		*Chlamydia trachomatis*		*Neisseria gonorrhoeae*	
		95% CI*	*P*-value	95% CI*	*P*-value
**Symptoms when tested**	Positive predict value	0.154 (0.027–0.404)	0.124	0.154 (0.027–0.404)	0.124
	Negative predict value	0.886 (0.830–0.929)	< 0.001	0.937 (0.892–0.968)	< 0.001
	Sensitivity	0.100 (0.017–0.278)	0.136	0.167 (0.030–0.431)	0.121
	Specificity	0.927 (0.878–0.961)	< 0.001	0.931 (0.884–0.963)	< 0.001
**Urine samples**	Positive predict value	0.857 (0.506–0.991)	< 0.001	0.800 (0.372–0.987)	< 0.001
	Negative predict value	0.905 (0.854–0.943)	< 0.001	0.941 (0.899–0.970)	< 0.001
	Sensitivity	0.273 (0.119–0.477)	0.004	0.286 (0.099–0.545)	0.018
	Specificity	0.993 (0.972–1.000)	< 0.001	0.994 (0.973–1.000)	< 0.001
**Anal samples**	Positive predict value	0.947 (0.788–0.997)	< 0.001	0.923 (0.703–0.995)	< 0.001
	Negative predict value	0.974 (0.941–0.992)	< 0.001	0.988 (0.962–0.988)	< 0.001
	Sensitivity	0.818 (0.627–0.940)	< 0.001	0.857 (0.621–0.975)	< 0.001
	Specificity	0.993 (0.972–1.000)	< 0.001	0.994 (0.973–1.000)	< 0.001

*CI: Confidence Interval; *P*-value refers to χ2 test

In Table 5 is possible to check that the proposed model fitted the data well. Non-significant direct hypothetical trajectories were removed from the complete theoretical model, which was re-assessed to obtain a statistically parsimonious model that is also suitable for the data.

**Table 5:** Summary of fit indices for full and parsimonious models

Model	χ2/df ratio	GFI	CFI	SRMR	RMSEA
Full theoretical model	0.723	0.990	1.000	0.0295	0.000
Parsimonious model	0.804	0.973	1.000	0.0486	0.000
Ideal value	< 3.0	≥ 0.95	≥ 0.95	≤ 0.08	≤ 0.06

χ2/df ratio Chi square and degrees of freedom ratio; GFI: Goodness of fit statistics; CFI: comparative fit index; SRMR: standardized root-mean-squared residual; RMSEA: root-mean-square error of approximation

**Fig. 3:**
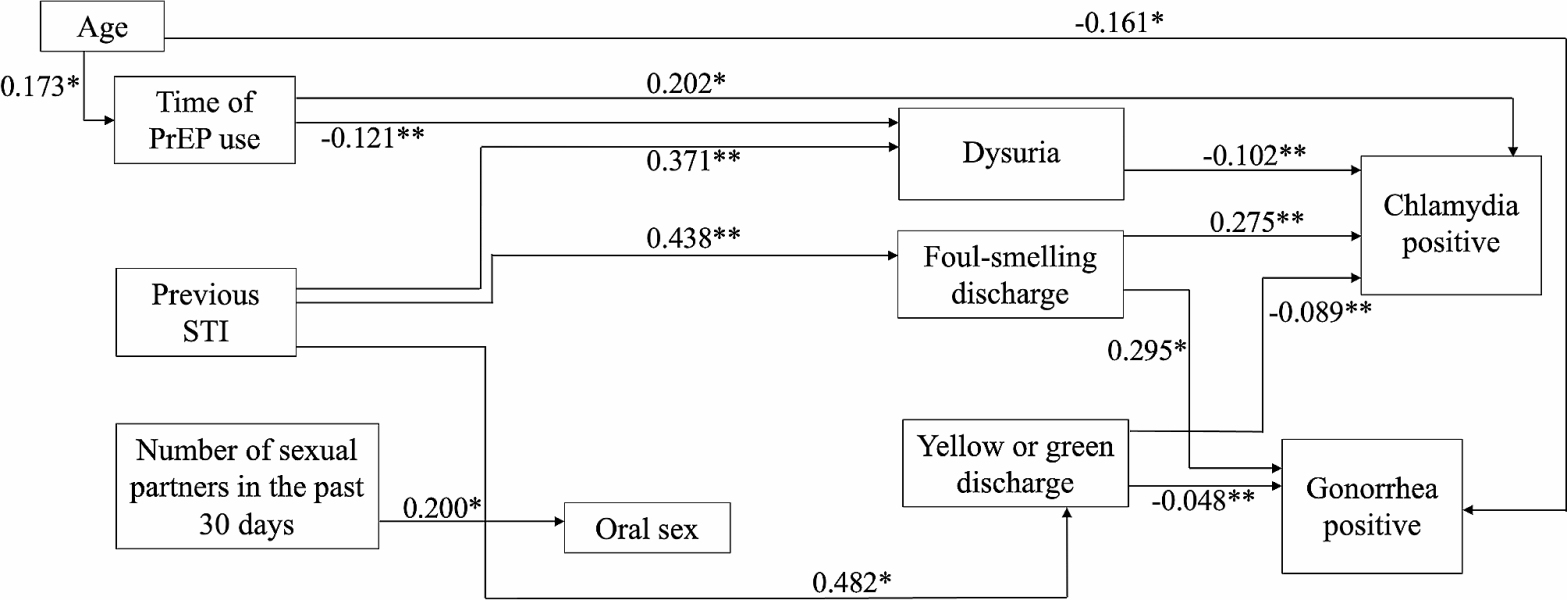
Parsimonious model. Statistically significant direct relationships in the parsimonious model, including standardized β-coefficients for each path. Notes: *p < 0.1, **p < 0.05, ***p < 0.01

Removing the nonsignificant paths did not modify the original model. The final model was accepted, and the path analysis results are presented below in the Fig. 3.

Practicing anal sex and anilingus were not associated with any other variable in the full model and thus were removed from the parsimonious model. In the parsimonious model, higher number of sexual partners was linked to oral sex practice (β= 0.0200, p= 0.035). Gonorrhoea was directly linked to lower age (β= -0.161, *p* = 0.001). Duration of PrEP use was directly associated with CT infection (β = 0.202; *p* = 0.042) and inversely associated with dysuria (β= -0.121, *p* = 0.009). The latter was linked to lower likelihood of chlamydia detection (β= -0.102, *p* = 0.001). Lower occurrence of yellow-green secretion was linked to detection of CT (β= -0.089, *p* = 0.005) and NG (β= -0.048, *p* = 0.002) infections. Foul-smelling discharge was directly associated with CT (β = 0.275, *p* = 0.004) and NG (β = 0.295, *p* = 0.037) infection.

It was observed na indirect association between age and dysuria (β= -0.021, *p* = 0.016). The NG was indirected connected with the age (β= -0.001, *p* = 0.378), previous STI (β = 0.120, *p* = 0.191) and with time of PrEP use (β= -0.004, *p* = 0.589). Further, the presence of CT had na indirect relation with age (β = 0.037, *p* = 0.030), with previous STI (β = 0.40, *p* = 0.353) and with time of PrEP use (β = 0.012, *p* = 0.006).

## Discussion

This study evaluated CT/NG screening at two anatomical sites. We made a pathway analysis and the paths between the three variables representing clinical symptoms (malodorous, discharge and dysuria) and positive detection for Chlamydia and Gonorrhoea were retained in the model according to the central hypotheses of the study. The evidence found in this study provides relevant information to improve sexual health outcomes, as well as to demonstrate the need for the integration of CT/NG testing into PrEP services and to achieve the United Nations Sustainable Development Goals that propose the control of the double epidemic of HIV and STI in the world [12].

Our main findings suggested that symptoms are a bad indicator of CT/NG infection and the screening must be done in more than one anatomical site, since most of the positive results would be missed if only urine were tested. In the case of testing only one anatomical site, specifically the urethra, the CT/NG prevalence would be underestimated. Our findings are consistent with findings from previous studies that showed a great amount of asymptomatic extragenital CT/NG infections [13, 14] and that multisite testing improves detection rates of these pathogens, interrupting transmission and preventing complications [15, 16].

The most striking difference was between the anatomical sites. Pathogen detection was five times higher in anal samples than in urine. The urine showed low sensitivity for CT/NG detection. This data may be related to the fact that in men only 10% of urethral NG infections are asymptomatic [9]; So, men with urethral NG infection tend to seek treatment quickly. Furthermore, it may also be related to more receptive than insertive anal sex of our participants. Studies that identified NG in oropharyngeal and anorectal samples showed that the penis is not the only site involved in gonorrhoea transmission [16, 17], especially in cases of sexual practices that alternate oral-anal sex [18]. Therefore, the two or multiple-site testing is indispensable to improve the screening of these pathogens, as well as detect asymptomatic infections.

We identified that dysuria and the presence of yellow or green discharge, by itself, had a lower chance of affecting the outcome. The literature shows that, in fact, these infections can be asymptomatic for a long period of time, especially in cases of extragenital infection [3, 9, 13, 14]. Furthermore, our results contribute with evidence that testing different anatomical sites improves the screening of CT and NG infections even when asymptomatic [15, 19]. The WHO encourages multi-site testing for bacterial STIs even in the absence of symptoms, as part of care for people at considerable risk of acquiring HIV [20]. In Brazil, the Ministry of Health recommends testing PrEP users through urine or genital secretions every six months or in case of symptoms [5]. However, testing for these pathogens is still insufficient and there is an inconsistency between clinical practice and the national protocol [7]. This is problematic as clinical decisions for the treatment of CT/NG are being guided by a criterion of poor effect on outcome. Therefore, the expansion of PrEP must also be accompanied by the expansion of PCR tests for STIs in Brazil at different levels of health care.

The presence of foul-smelling discharge was positively associated with CT (β = 0.275, *p* = 0.004) and NG (β = 0.295, *p* = 0.037) infection. The literature shows that purulent secretion is prevalent in men with NG urethral infection [9, 21]. However, our sample was predominantly composed of MSM, whose pathogens were identified only in the anorectal region with greater frequency for CT. The presence of enterobacteria in this anatomical site may contribute to the occurrence of malodorous. However, 7.6% participants had any STI symptom when tested and only 2.3% had a positive PCR result of CT and 1.8% of NG infection. Based on this, our finding might be associated with a possible co-infection of any other STI, since they share the same transmission path.

Increasing PrEP usage time increases the chance of CT detection. This may be related to a possible reduction in condom use after adopting PrEP, as previously described in another study [22]. On the other hand, our finding may be related to the fact that PrEP follow-up benefits people for testing and early treatment of STIs [23]. This reveals the importance of PrEP services emphasising existing STI control strategies [17], in addition to combined prevention strategies that consider behavioural interventions such as counselling and discussion about the risk of other STIs under condomless intercourse.

A modelling study showed that around 40% of CT/NG infections would be prevented over the next decade if 40% of PrEP-eligible men took PrEP and were tested twice a year for STIs. Biannual screening linked to PrEP care treated 17% more asymptomatic infections, 16% more rectal infections, and was associated with a decline of 67% in combined STI incidence among all MSM. However, increasing the frequency of screening for these STIs to every three months would result in an additional 50% reduction in STI incidence [23].

Despite the high effectiveness of diagnostic tests, the high cost of laboratory supplies makes their feasibility difficult in developing countries. So, studies have shown that ‘pooling’ multiple-site samples could be a cost-saving strategy for CT/NG screening with minimal decrease in sensitivity. With this technique, collected samples are pooled into the same vial. It increases the chance of screening the pathogen while saving reagents and laboratory supplies [15, 19]. Therefore, this might be a meaningful strategy for countries with a public health system intending to carry out large-scale testing to control the epidemiological situation of their populations.

In summary, our findings suggest that including CT/NG testing periodically in PrEP follow-up allows for the timely screening and diagnosis of these pathogens, even when asymptomatic. In this way, we support that PrEP benefits go beyond HIV prevention, as it enables access to testing and treatment for other infections that can occur asymptomatically and harm the sexual health of its users and their sexual networks. Further, we strongly encourage health authorities to enhance efforts to make molecular biology methods available homogeneously at different levels of health services to improve the screening and early treatment of these infections.

We did not collect pharyngeal samples, which limits our approach to multi-site testing. However, our two-site testing data proved to be relevant for diagnostic criteria, especially concerning anal samples. Our study should be interpreted with some caution. Firstly, our sample was reached by convenience, so the presented findings cannot be extrapolated to populations that are different from the one studied according to the inclusion criteria. However, our participants’ profile is the same as the official data of PrEP users in Brazil presented by the Ministry of Health [24]. Further, we did not specify the sex activities, so we cannot state if most sex activities happened in the receptive, active or both positions. Moreover, we did not screen other pathogens which may interfere with the symptoms. Finally, data about alcohol consumption and drug use might be underestimated due to recall bias since these data were collected considering the last six months.

So, we suggest further studies with multisite testing among different populations for a better understanding of the prevalence of CT/NG in real-world settings. These studies may reveal a higher prevalence of CT/NG than estimated in the general population and among its subgroups, impacting public policies focused on combating the transmission cycle of these pathogens.

## Conclusion

In our findings showed that the symptoms were a bad indicator of CT and NG infection, and the screening must be done in multiple anatomical sites since most of the positive results would be missed if only urines were tested. In the case of testing only one anatomical site, specifically the urethra, the CT/NG incidence and prevalence would be underestimated. Expanding the supply of these tests facilitates the shift from syndromic to causal treatment, as well as improving the screening of asymptomatic cases that contribute to the transmission cycle. Finally, the PrEP follow-up benefits people for testing and early treating STIs.

## Data Availability

The data that support the findings of this study are available from M.A. and R.K.R. but restrictions apply to the availability of these data, which were used under license for the current study, and so are not publicly available. Data are however available from the authors upon reasonable request and with permission of Marcela Antonini and Renata Karina Reis.
